# Ipsilateral distal femoral and proximal tibial epiphyseal growth plate injury: a case report

**DOI:** 10.1186/1752-1947-7-146

**Published:** 2013-05-31

**Authors:** Deniz Gulabi, Mehmet Erdem, Guven Bulut, Cem Coskun Avci, Murat Asci

**Affiliations:** 1 Dr. Lütfi Kırdar Kartal Training and Research Hospital, Semsi Denizer Cd E 5 Karayolu Cevizli Mevkii 4 Kartal, İstanbul 34890, Turkey; 2 Sakarya Üniversitesi Tıp Fakültesi Ortopedi ve Travmatoloji Bilim Dalı, Esentepe Campus, Sakarya 51487, Turkey; 3 Ümraniye Training and Research Hospital, Kazım Karabekir, Ümraniye, İstanbul 34000, Turkey; 4 Department of Orthopaedics and Traumatology, Tokat Devlet Hastanesi, Tokat 60100, Turkey

**Keywords:** Knee, Growth plate, Epiphysiolysis, Surgical treatment

## Abstract

**Introduction:**

Both the isolated distal femoral epiphysiolysis and the isolated proximal tibial epiphysiolysis are the least common epiphyseal injuries. Even though they are uncommon, they have a high incidence rate of complications.

**Case presentation:**

We present a case with Gustilo-Anderson grade 3b open and Salter-Harris type 1 epiphysiolysis of the distal femur and proximal tibia caused by a farm machinery accident. The patient was a 10-year-old boy, treated by open reduction and internal fixation.

**Conclusion:**

Although distal femoral and proximal tibial growth plate injuries are rarely seen benign fractures, their management requires meticulous care. Anatomic reduction is important, especially to minimize the risk of growth arrest and the development of degenerative arthritis. However, there is a high incidence of growth arrest and neurovascular injury with these type of fractures.

## Introduction

Isolated distal femoral epiphysiolysis, and the isolated proximal tibial epiphysiolysis are both very rarely seen [1,2]. Distal femoral and proximal tibial epiphysiolysis comprise 3% and 0.5% of all epiphysiolysis, respectively [3,4]. Even though they are uncommon, they have a high incidence rate of complications [1,2]. They are mostly caused by traffic accidents, sports trauma, and horse riding accidents [5].

In our review of English language medical literature, ipsilateral distal femoral and proximal tibial epiphysiolysis have rarely been encountered. Our patient was the first case with Gustilo-Anderson grade 3b open and Salter-Harris (SH) type 1 epiphysiolysis caused by a farm machinery accident.

## Case presentation

**Figure 1:**
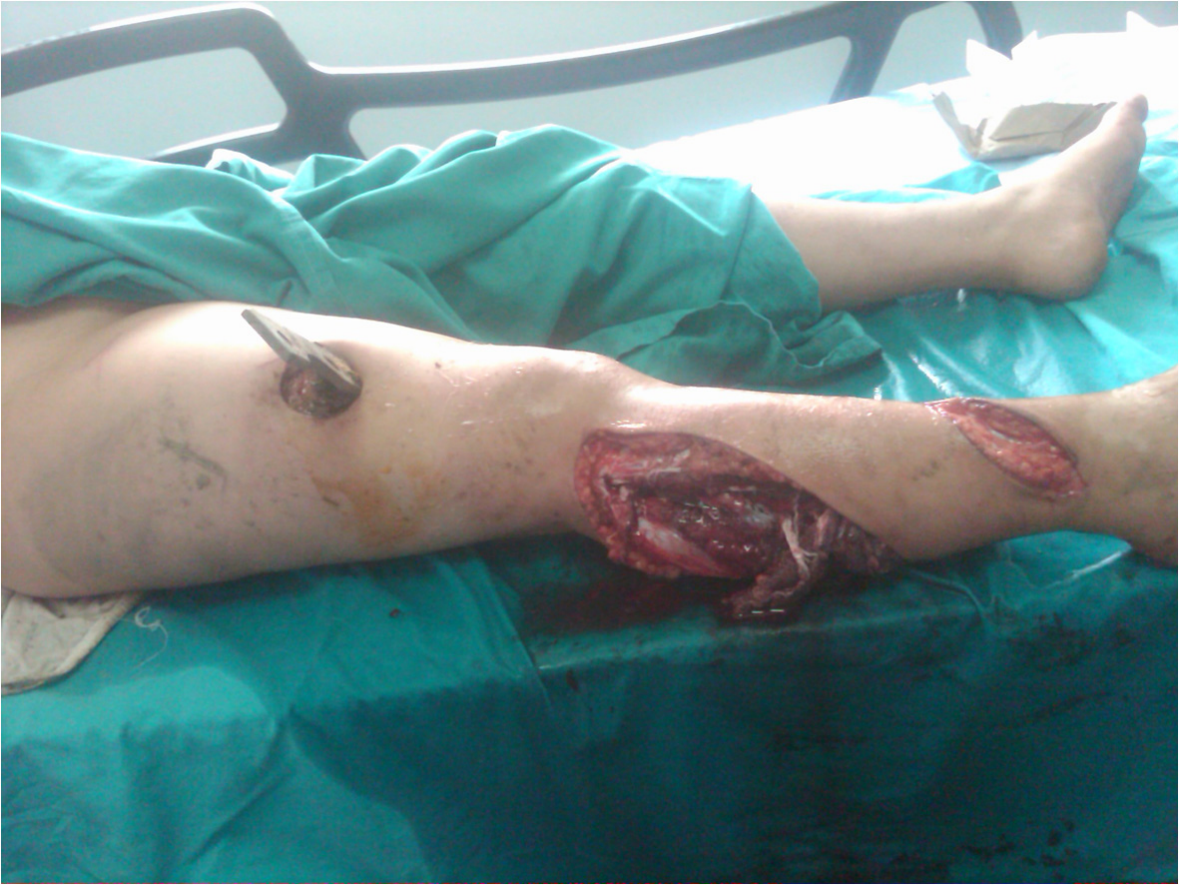
Immediate view of the patient, who had a farm machinery accident, in the emergency room.

A 10-year-old, 50kg boy presented with a severe injury on his right knee as a result of a farm machinery accident. The mechanism of the injury was a hyperextension and torsional force around the knee. On clinical examination, a 5×10cm skin defect was seen on the anterolateral aspect of the proximal end of the right leg (Figure 1). His lateral compartment muscles and peroneal nerve were severely damaged. The vascular status of the involved leg was normal; the arteria dorsalis pedis, arteria tibialis posterior and arteria poplitea were palpable, and capillary filling of the foot was present. In addition, no vascular problems were detected by the Doppler ultrasound of the involved limb.

**Figure 2:**
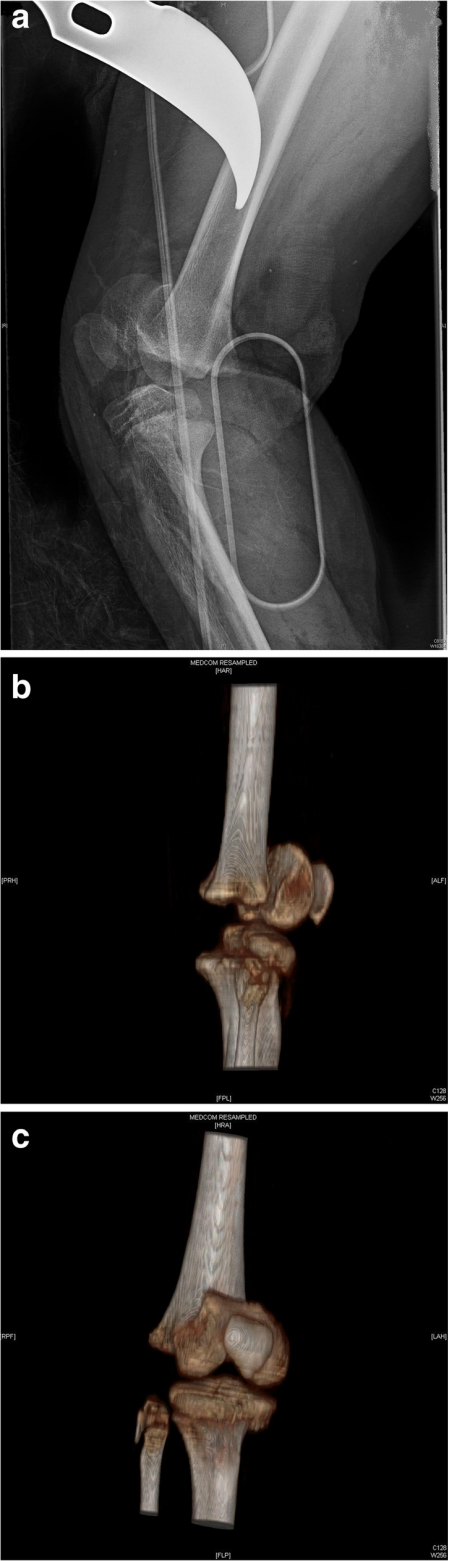
**Preoperative radiological images demonstrating the severe right knee injury. (a)** Lateral radiograph of the knee. **(b)** Sagittal computed tomography (CT) reconstruction showing the physeal fractures. **(c)** Coronal CT three dimensional reconstruction.

After the debridement and irrigation of his wound, direct radiographic and computed tomography (CT) examinations determined a right distal femur type 1, and right proximal tibia type 1 epiphysiolysis and a right proximal fibula Salter-Harris type 2 fracture (Figure 2a, b, c). We applied prophylaxis to prevent the development of tetanus and gaseous gangrene; we started gentamicin (80mg intramuscular for three days), metronidazole (500mg intravenous for three days) and first-generation cephalosporin (cephazolin sodium 1000mg intravenous for three days).

Surgical treatment of the patient was performed on the day of presentation. The patient was placed in a supine position under general anesthesia. His right knee was approached through a 3cm-long lateral longitudinal incision at the level of the distal femoral physis. Skin, subcutaneous tissue, fascia lata, and vastus lateralis were dissected away, and periosteum was elevated. By flexing the knee 90°, the posteriorly displaced distal femoral epiphysis was reduced with a blunt periosteal elevator. Fixation was achieved by using two crossing 4.0mm cannulated screws from distal to proximal direction. After debridement and irrigation of the large skin and soft tissue lesion on the lateral aspect of the proximal tibia, a 3cm longitudinal incision, proximal to the skin defect at the level of the proximal tibial physis, was performed. Then, skin, subcutaneous tissue, and periosteum were elevated. While the knee was positioned at 90° flexion, proximal tibial epiphysis was reduced, and fixation was achieved by using two crossing 4.0mm cannulated screws.

**Figure 3:**
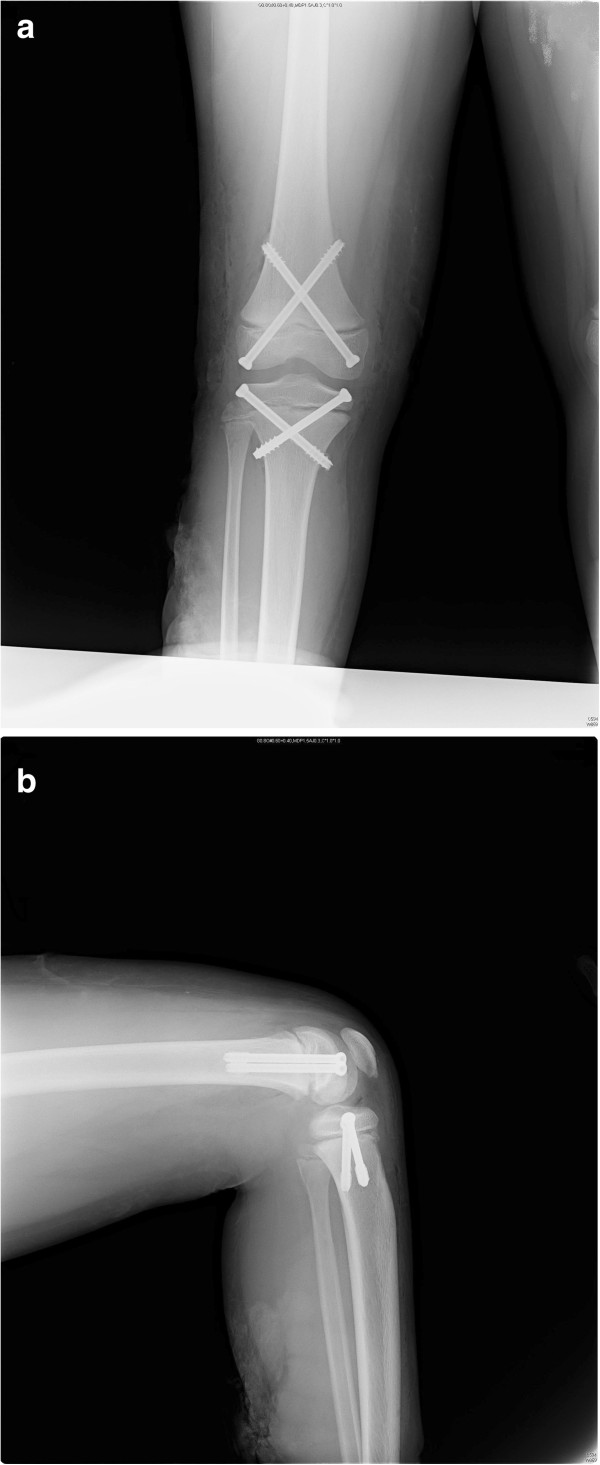
**Radiological images of the patient at three weeks after the operation of his right knee demonstrating anatomic reduction. (a)** Anteroposterior and **(b)** lateral radiographs of the knee at three weeks after fixation.

Anatomic reduction was observed on postoperative plain radiographs. The consultant plastic surgeon recommended to follow up with a wet dressing for a while. The peroneal nerve was not intervened, and an ankle-foot orthosis (AFO) was prescribed to hold the ankle in the neutral position. Isometric quadriceps strengthening and knee range of motion (ROM) enhancing exercises were begun at postoperative day two. After 20 days, following the maturation of satisfactory granulation tissue, skin grafting was performed. Then, the patient was discharged with a hinged brace locked in full extension for ambulation (Figure 3a, b). The brace was removed at the sixth postoperative week, and full weight bearing was initiated on the involved extremity with good quadriceps strength for safe ambulation. The hardware was removed six months after the operation. The patient achieved full knee ROM, while the peroneal nerve lesion persisted.

**Figure 4:**
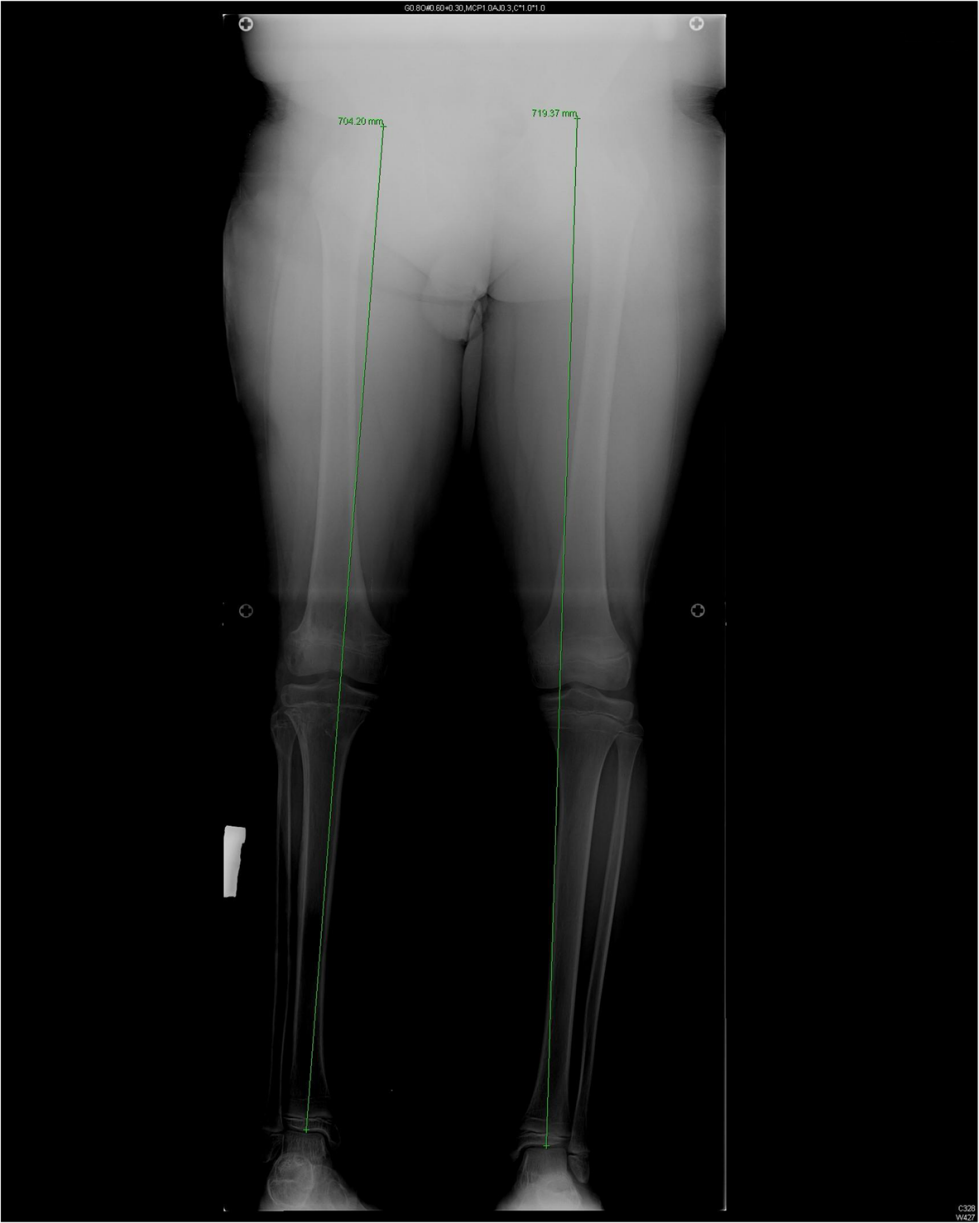
**Orthorontgenogram of the patient at 18 months after fixation.** The dotted line is the mechanical axis deviation (MAD).

At the 18-month follow-up examination, the patient’s injured extremity was 1.4cm shorter than the contralateral limb. The lower limb shortening of the injured leg was 1.2cm from the femur; 0.2cm was due to the tibia. However, no angular deformity was detected on the orthorontgenograms of both lower extremities obtained in the standing position (Figure 4). Knee ROM was 0° to 130°. There was no quadriceps atrophy, and strength was comparable to that of the contralateral side with a Lysholm knee score of 90 [3]. The patient had no knee pain and edema. He is currently using an AFO that holds the ankle in the neutral position. At the 18-month follow-up examination, the deep peroneal nerve lesion persisted, but no superficial peroneal nerve injury was detected.

## Discussion

Distal femoral epiphysis provides 70% of the longitudinal growth of the femur, and 40% of the overall growth of the lower extremity. Skeletal growth at the distal femoral physis is the fastest of all physis [1,2]. Distal femoral epiphysiolysis is a rarely seen injury with frequently seen complications [1,2]. Among all of the most observed epiphysiolysis, it is the most common growth plate injury in children [2]. It constitutes 5% of all epiphyseal fractures [3,4].

Distal femoral epiphysis is present at birth, and forms both femoral condyles. It fuses with metaphysis in girls at 14 to 16, and in boys at 16 to 18 years of age [4,5]. Distal femoral epiphysis forms the proximal insertion point of the gastrocnemius. Distal femoral epiphysiolysis mostly occurs as a consequence of traffic accidents, sports (especially horse riding) injuries, and falls from a height [5]. Epiphyseal fractures were first mentioned by Ollier in 1867 [6]. They are classified according to the Salter-Harris classification. Salter-Harris type 2 is the most encountered form [1,5-7]. In 1894, Hutchinson reported that rotation or traction of the femur was the pathogenic mechanism of the femoral epiphysiolysis [2,6].

The most frequently observed complications of distal femoral epiphysiolysis are growth disturbance, angular deformities, restricted ROM, instability, and neurovascular problems [2,3,8,9]. The severity of the damage to the growth plate may be related to several factors; including age, high-energy trauma, type of fracture, degree and direction of displacement, and violation of the physis by pins [3].

Growth arrest is the most observed complication, which has been shown to account for 40% of cases [3]. Clinically, poor outcomes are defined as a leg length discrepancy of ≥1.5cm and varus, valgus or flexion deformity of ≥10° [2,8]. Growth arrest is frequently seen, especially in SH type 4 fractures. Growth arrest can occur as a result of direct physeal injury, epiphyseal bone bridge formation or nonanatomical reduction, which can be demonstrated by magnetic resonance imaging (MRI) [1]. Basener *et al.* reported that growth arrest had occurred in 64% of SH type 4, 49% in SH type 3, 58% in SH type 2, and 36% in SH type 1 fractures [2]. Extreme displacement and advanced SH type are predisposing factors for growth arrest [1,2].

Several studies have shown that incidence of growth disturbance after distal femoral physis disruption is high, and usually results in leg length discrepancy, angular deformity, or both [1,7]. The amount of displacement is calculated as follows: grade 1, <1/3 of the bone diameter; grade 2, 1/3 to 2/3 of the bone diameter; grade 3, >2/3 of the bone diameter; grade 4, comminuted fractures [1]. Growth disturbance in the extremely displaced and nondisplaced fractures was reported at 65% and 31%, respectively [2]. Significant impact of the amount of displacement on the development of complications has been demonstrated statistically [1,2]. There is a correlation between poor outcomes and age, in that undesirable outcomes after distal femoral physeal fractures are more common in younger patients [2,8,9].

The ossification center of the proximal tibial epiphysis appears between postnatal months one and three, and it enlarges to the periphery irregularly. Tuberositas tibia appears as an extension of the proximal tibial epiphysis at the 13th gestational week and, at approximately eight years of age, a second ossification centre develops distal to the tibia. When the adolescent is nearly 17 years old, these two ossification centers fuse. The proximal tibial physis contributes 55% of the length of the tibia, and 25% of the entire length of the lower extremity (5,10). Since the proximal tibia does not have any insertion point for ligaments, it is not exposed to varus or valgus stresses exerted via ligaments, and consequently proximal tibial epiphyseal fractures are very rarely seen entities, constituting only 0.5% of all epiphyseal fractures [5,10-12]. They frequently occur after a violent trauma, and especially in boys aged 12 to 14 years [10,11]. Usually, SH type 2 injuries are observed [5,12]. Among its many complications, angular deformity in the form of genu recurvatum and relative asymmetric shortness of the affected extremity as a result of earlier closure of the growth plate are mostly seen [10,11]. The popliteal artery courses near the proximal tibial epiphysis, and so it can be traumatized especially due to impingement of the distal fragment of the fractured bone [5].

The management of these fractures consists of closed reduction and application of an above-the-knee cast in SH types 1 and 2 injuries. However, if a closed reduction is impossible or the fracture is unstable, surgical treatment is indicated. For SH type 3 and 4 fractures open reduction and internal fixation is recommended. Since arterial occlusion is the most threatening complication of these interventions, arterial circulation should always be checked carefully after the reduction.

## Conclusion

Although distal femoral and proximal tibial growth plate injuries are rarely seen benign fractures, their management requires meticulous care. Anatomic reduction is important, especially to minimize the risk of growth arrest and the development of degenerative arthritis. The patient and his/her next-of-kin should be informed about the potential limb length discrepancy, and/or angular deformities that might happen in the future. A CT scan evaluation is essential for recognition of the fracture pattern and preoperative planning. These patients should be followed up until their skeletal maturity is complete.

## Consent

Written informed consent was obtained from the patient’s next-of-kin for publication of this manuscript and any accompanying images. A copy of the written consent is available for review by the Editor-in-Chief of this journal.

## Competing interest

The authors declare that they have no competing interests.

## Authors’ contributions

The patient was under the care of ME. ME and MA surgically operated on the patient. CCA analyzed the data. DG wrote the manuscript. GB made additions to the manuscript. All authors reviewed and approved the final manuscript.
